# A Case of Cardiobacterium hominis Endocarditis Manifesting as Isolated Renal Infarction: Clinical Insights and Novel Presentation

**DOI:** 10.7759/cureus.91880

**Published:** 2025-09-09

**Authors:** Jotaro Yamamoto, Chinatsu Komiyama, Mizuki Haraguchi, Masamichi Imai, Takahide Kodama

**Affiliations:** 1 Cardiovascular Center, Toranomon Hospital, Tokyo, JPN; 2 Department of Infectious Diseases, Toranomon Hospital, Tokyo, JPN; 3 Department of Radiology, Toranomon Hospital, Tokyo, JPN; 4 Cardiovascular Center, Toranomon hospital, Tokyo, JPN

**Keywords:** cardiobacterium hominis, infective endocarditis, intravascular ultrasonography, renal artery infarction, successful revascularization

## Abstract

A 47-year-old woman presented with renal infarction due to an unknown etiology. Intravascular ultrasonography (IVUS) revealed emboli rather than dissection, guiding successful renal artery intervention that resolved her abdominal pain and preserved renal function. Blood culture revealed *Cardiobacterium hominis*, and echocardiography confirmed infective endocarditis with vegetation on the mitral valve. Antimicrobial therapy effectively controlled the infection without worsening mitral regurgitation or embolism. This case highlights the role of IVUS in differentiating emboli from dissection, demonstrating the benefits of revascularization in renal infarction and representing a rare instance of renal infarction solely caused by *C. hominis*.

## Introduction

Renal infarction is a rare but serious condition characterized by the occlusion of the renal arteries or their branches, leading to ischemia and necrosis of the kidney tissue. This condition results from a disruption in the blood supply to the kidneys, which can occur due to various causes, including embolisms from the heart and proximal aorta, abdominal trauma, and intrinsic pathology of the renal artery [1]. Cardioembolic events are the most common cause of renal infarction, with atrial fibrillation (AF) being the predominant underlying condition, accounting for 40-48% [1-3]. However, according to the report by Oh et al., infective endocarditis (IE) is a much rarer cause, responsible for only about 3.4% of renal infarction cases [3], so diagnosing the underlying cause can be challenging. Furthermore, renal dysfunction is a common complication associated with renal infarction. There are certain situations where endovascular intervention may be recommended to treat renal infarction, but the risks and benefits of the procedure should be carefully weighed.

*Cardiobacterium hominis* is a gram-negative, facultatively anaerobic bacillus that is part of the HACEK (*Haemophilus *species, *Aggregatibacter* (formerly* Actinobacillus*) species, *Cardiobacterium hominis*, *Eikenella corrodens*, and *Kingella* species) group of bacteria, which causes IE [4,5]. The HACEK group of organisms is notable for being a rare cause of IE, accounting for approximately 1.3-3% of IE cases. HACEK endocarditis progresses slowly, often leading to delays in diagnosis. Although the cumulative mortality rate is relatively low (6% in HACEK vs. 39% in non-HACEK endocarditis) [6], the infection can result in the formation of large vegetations on heart valves, which increases the risk of significant embolic events. Prior reports indicate that renal infarction in *C. hominis* infective endocarditis is extremely rare and almost always accompanied by additional embolic events such as cerebral or splenic infarctions [4]. We report a case of IE caused by *C. hominis*, presenting solely with renal infarction. The patient was successfully treated with early endovascular intervention and antibiotic therapy without major complications.

## Case presentation

Initial presentation

A 47-year-old woman without a known history of cardiovascular disease presented to the emergency department of an outside hospital with sudden right flank pain. Her medical history included two cesarean sections and a diagnosis of polycystic ovary syndrome 20 years ago; she had also been diagnosed with periodontal disease in the recent past but had not yet received treatment. The patient reported no observation of fever prior to presentation; however, a fever of 37.9℃ was noted upon admission. A contrast-enhanced CT (CECT) scan of the abdomen suggested an occlusion just distal to the bifurcation of the right renal artery, and subsequent renal angiography confirmed a 99% stenosis of the lesion. The patient was therefore admitted to the referring hospital with a diagnosis of renal infarction. Continuous heparin infusion and symptomatic treatment were initiated.

**Figure 1:**
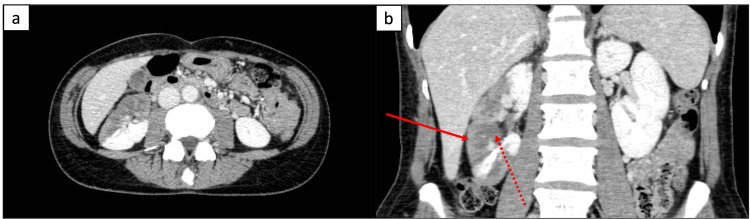
Contrast-enhanced CT of the abdomen showing right renal infarctions; enhancement of the renal cortex was preserved during the late phase. (a) transverse section, (b) coronal section.

Despite no deterioration in renal function, the patient was transferred to our hospital for revascularization three days after the event due to persistent right flank pain and lack of improvement. The patient was afebrile prior to admission, with a maximum recorded temperature of 37.5℃, but presented with a low-grade fever of 37.9℃ on admission. Her heart rate was 76 beats per minute, blood pressure of 128/64 mmHg, and oxygen saturation of 97% on room air. Physical examination revealed a 3/6 holosystolic murmur at the cardiac apex, and there was significant tenderness on palpation of the right flank. Laboratory findings showed a white blood cell count of 9100/μL (slightly elevated), lactate dehydrogenase (LDH) of 338 IU/L (slightly elevated), C-reactive protein (CRP) of 3.64 mg/dL (elevated), and brain natriuretic peptide (BNP) of 279.9 pg/mL (elevated). Her serum creatinine (0.43 mg/dL) was within normal limits. A CECT scan of the abdomen (Figure 1) showed a perfusion defect in the upper lateral aspect of the right kidney, but enhancement of the renal cortex was preserved during the late phase.

Revascularization procedure

**Figure 2:**
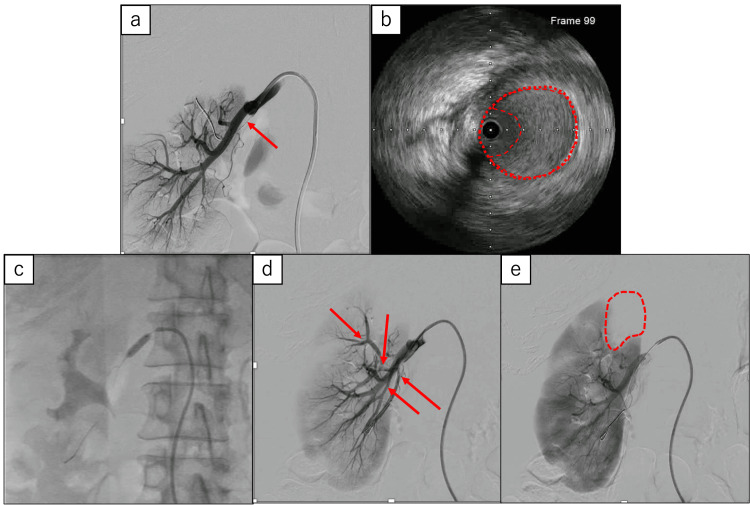
Renal angiography before revascularization reveals a complete occlusion of the ventral branch. (a) A guide wire is passed through the branch (arrow); (b) Intravascular ultrasound (IVUS) reveals the presence of extensive thrombus within the vessel lumen, and the vessel wall structure was preserved; (c) Balloon dilation of the proximal portion of the right renal artery; (d) Renal angiography following revascularization shows the four recanalized branches (red arrows); (e) Angiography demonstrates non-revascularized parenchymal regions (area enclosed by red dashed line).

The persistence of colicky pain in the right flank and the preserved enhancement of the renal cortex on CECT indicated the suitability of revascularization. Renal angiography (Figure 2a) revealed complete occlusion of the ventral branch, resulting in a lack of perfusion to the upper third of the right renal parenchyma. Intravascular ultrasonography (IVUS) of the renal artery (Figure 2b) showed that the vessel structure was intact, with no evidence of renal artery dissection. However, the lumen was filled with thrombus, suggesting that the renal infarction was caused by an embolic event rather than an arterial dissection. So multiple branches supplying the upper part of the right kidney were dilated multiple times, both centrally and peripherally, using balloon catheters (Figure 2c). As a result, successful reperfusion was achieved in approximately 90% of the right renal parenchyma, confirming revascularization (Figures 2d-2e).

Clinical course and outcome

Following revascularization, the abdominal colic pain rapidly subsided. However, the patient experienced persistent low-grade fever in the 37 ℃ range. On day 3, two sets of blood cultures taken at the previous hospital four days earlier tested positive for gram-negative bacilli. Identification of isolated bacilli was tried by matrix-assisted laser desorption/ionization time-of-flight mass spectrometry (MALDI-TOF MS) (Microflex LT, flex control 3.4.135.0, MALDI Biotyper 4.1.1; Bruker Daltonics, Billerica, Massachusetts, United States), and *C. hominis* was identified with a score of 1.73. We finally confirmed it using 16S rRNA gene sequencing described previously [7].

**Figure 3:**
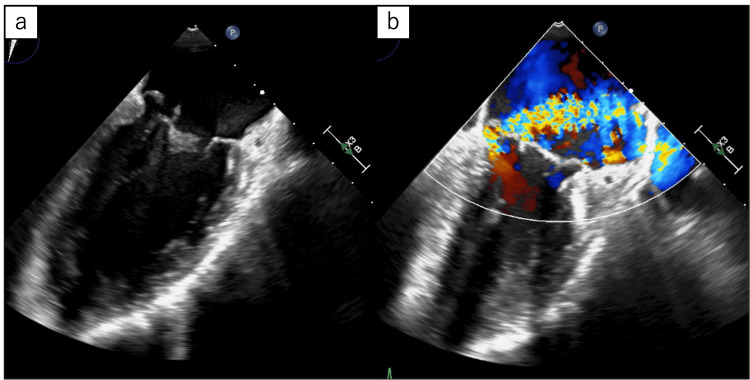
Transesophageal echocardiogram showing (a) several irregularly shaped hypoechoic masses approximately 4 mm in size on the mitral valve leaflets, and (b) moderate mitral regurgitation.

Cefepime was administered empirically and switched to ceftriaxone (2 g intravenously once daily) after the causative pathogen was identified as *Cardiobacterium* species. The isolate was susceptible to ceftriaxone (MIC: 1 μg/mL), cefepime (MIC: 1 μg/mL), and ampicillin (MIC: 0.25 μg/mL), based on antimicrobial susceptibility testing. The fever resolved quickly, but a transesophageal echocardiogram (Figure 3) revealed moderate mitral regurgitation (MR) and several irregularly shaped, hypoechoic masses approximately 4 mm in size on the native mitral valve leaflets, leading to a diagnosis of infective endocarditis according to the proposed modified Duke criteria [8].

This diagnosis was supported by two major criteria, positive blood cultures for* C. hominis* (a typical microorganism of IE) and echocardiographic evidence of vegetation, and one minor criterion, a vascular phenomenon (renal infarction). In accordance with clinical recommendations, anticoagulation with heparin was discontinued to reduce the risk of hemorrhagic complications. So, heparin was discontinued. CECT and brain MRI revealed no evidence of embolic findings other than in the kidney.

The patient experienced no worsening of MR and completed a four-week course of antibiotics before being discharged, in accordance with treatment guidelines for native valve IE. A renal ^99m^Tc-MAG_3_ scintigraphy was performed before discharge and indicated that the amount of uptake in the upper lateral portion of the right kidney in the perfusion phase was reduced compared to the left kidney, suggesting decreased renal function. However, in the secretion phase, the delayed uptake suggested that while there was a decrease in renal function, complete function loss did not occur.

## Discussion

In this report, we described a case of IE caused by *C. hominis*, diagnosed following a renal infarction. Revascularization contributed to the preservation of renal function, allowing the patient to be discharged without complications. This represents a valuable case in the diagnosis and management of this rare condition.

The treatment of renal infarction ranges from conservative management to surgical interventions. Revascularization is recommended in some situations [9,10]. In general, the timing of revascularization significantly affects its success in treating renal infarction. Early diagnosis is crucial for initiating revascularization therapy promptly, which optimizes renal function recovery. However, there have been several reported cases where renal artery revascularization performed more than 24 hours after the onset resulted in the recovery of renal function [11-13]. Ouriel et al. reported that in cases of embolic occlusion, revascularization performed even within six hours of onset does not necessarily lead to the recovery of renal function; in contrast, in cases of thrombotic embolism, renal function recovery may still be possible even if more than two weeks have passed since onset [14]. This suggests that factors other than the time of onset may play an important role in the outcome. In our case, CECT showed preserved cortical enhancement, suggesting the possibility of reversible renal infarction, and given the uncontrolled abdominal pain and the need for symptom improvement, we performed renal revascularization. As a result, renal function was preserved, and the abdominal pain resolved, indicating that renal revascularization was beneficial.

Angiography is the dominant imaging modality in revascularization; however, IVUS is also used as a modality for the management of renal infarction to enhance diagnostic accuracy and guide treatment strategies because it can accurately size vessels, determine the degree of stenosis, and characterize lesion morphology [15]. Ivanes et al. reported that performing IVUS in patients with renal infarction could facilitate a precise assessment of the underlying cause and contribute to modifications in the treatment strategy [16]. In our case, the use of IVUS was instrumental in assessing that the cause of the renal infarction was embolic rather than due to abnormalities in the vessel wall, proving to be highly valuable. To our knowledge, this is the first published case describing the use of IVUS in the diagnosis and management of septic renal embolism.

The HACEK group of bacteria has been well recognized as a cause of IE, and it is characterized by frequent colonization of the oropharynx, slow growth, and enhanced growth in the presence of carbon dioxide, which leads to the delay of the diagnosis [17]. For organisms in the HACEK group, it typically takes five to seven days to identify the causative bacteria from blood cultures [18]. In our case, it took three days to detect gram-negative bacilli from blood cultures, and it is not easy to identify *C. hominis* using in-house materials, which suggests that initiation of treatment may be delayed compared to IE caused by gram-positive cocci. The use of MALDI-TOF MS, followed by 16S rRNA gene sequencing, proved essential for the accurate identification of *C. hominis*, a fastidious and slow-growing organism. These advanced microbiological tools are particularly valuable in cases of culture-positive gram-negative bacteremia where conventional methods may fail or delay diagnosis.

Additionally, the patient had a history of untreated periodontal disease. *C. hominis* is a commensal organism in the oral cavity, and approximately 44% of infective endocarditis cases caused by *C. hominis* have a history of prior oral infection or dental procedures [19]. Untreated periodontal disease can compromise the oral mucosal barrier, facilitating the translocation of oral bacteria like *C. hominis* into the bloodstream and increasing the risk of bacteremia and subsequent endocarditis. The HACEK group has several distinct clinical characteristics compared to the non-HACEK group. The median age of HACEK endocarditis cases is 47.4 years, which is significantly lower than the age of non-HACEK endocarditis cases (60.5 years) [6]. Compared to non-HACEK endocarditis, HACEK endocarditis tends to be subacute with a median duration of about 13 weeks of symptoms before diagnosis, so it is more prone to have a larger vegetation at diagnosis [19]. That means HACEK endocarditis has a higher probability of vascular and immunological manifestations, including strokes, due to increased embolization risk, which is consistent with our case. On the other hand, the HACEK group is more likely to be acquired from the community, so HACEK endocarditis has a better prognosis than non-HACEK endocarditis. Chambers et al. reported that the cumulative mortality rate was 6% in HACEK patients compared to 39% in non-HACEK IE patients [6].

While the prognosis for HACEK endocarditis is generally favorable, the risk of severe embolic events due to delayed diagnosis remains high, making early diagnosis and treatment essential. Particularly in cases like ours, where IE presents solely with renal infarction, there is a likelihood of delayed diagnosis due to its rarity. According to a review of case reports on IE caused by *C. hominis*, only one out of 21 cases involved renal infarction [4]. This appears to be the first reported case of *C. hominis* IE presenting solely with renal infarction. We successfully managed this unique case with early diagnosis and treatment, resulting in discharge without complications.

## Conclusions

This case illustrates several important clinical lessons. Isolated renal infarction can be the initial manifestation of IE caused by HACEK organisms such as *C. hominis*; so, clinicians should consider infective endocarditis in cases of unexplained renal infarction, especially in patients with risk factors such as periodontal disease. This report also shows that IVUS may aid in distinguishing embolic occlusion from arterial dissection. Finally, it is seen that early revascularization, even several days after onset, may contribute to symptom relief and preservation of renal function.
